# Safety and Feasibility of Salvage Endoscopic Combined Intrarenal Surgery in Embolized Kidney

**DOI:** 10.1089/cren.2016.0069

**Published:** 2016-07-01

**Authors:** Alfonso Benincasa, Federico Nicolosi, Giovanni Lughezzani, Nicolò Maria Buffi, Paolo Casale, Rodolfo Hurle, Massimo Lazzeri, Pasquale Cardone, Giorgio Guazzoni, Alberto Saita

**Affiliations:** ^1^ Department of Urology, Vittorio Emanuele Hospital, Catania, Italy.; ^2^ Department of Urology, Humanitas Clinical and Research Center, Humanitas University, Rozzano, Italy.

## Abstract

***Background:*** Although endoscopic combined intrarenal surgery (ECIRS) is well established as primary approach to complex lithiasis, no evidences are still available on its use in salvage context.

***Case Presentation:*** A male patient, of 55 years of age, underwent many unsuccessful surgical procedures to treat large and multiple right kidney stones, including percutaneous nephrolithotomy (PCNL). The latter was complicated by severe postoperative hemorrhage, managed with super-selective renal artery embolization (SRAE). Therefore he came to our institution to achieve a complete resolution of the urolithiasis. Preoperative evaluation included CT scan and renal scintigraphy to establish kidney and stone morphologic features and residual renal function. Salvage ECIRS was performed and postoperative assessment showed a complete resolution of lithiasis and absence of renal function impairment.

***Conclusion:*** To our knowledge, this is the first case of salvage ECIRS reported in literature after previous failed PCNL. Even after SRAE, this procedure appears as safe and as efficacious as standard salvage PCNL when performed by experienced hands.

## Introduction and Background

Endoscopic combined intrarenal surgery (ECIRS) is well established as a minimally invasive procedure for treatment of large, complex, and multiple urolithiasis, but at the moment there is no evidence of its use in salvage setting. Only one study is now available in literature focusing on safety and the effectiveness of salvage percutaneous nephrolithotomy (PCNL) after a previous unsuccessful PCNL. We would like to report the first case of salvage ECIRS performed in embolized kidney and its feasibility.

## Case Presentation

### Clinical history

A 55-year-old male came to our institution with multiple and complex right renal stones in embolized kidney for previous PCNL failure complicated by intraoperative bleeding that forced the interruption of the procedure and postoperative hemorrhage, managed with selective renal artery embolization (SRAE). He also revealed right lumbar pain, flank irradiation, and moderate hydronephrosis. His medical history included arterial hypertension, familial hypercholesterolemia, and sick sinus syndrome managed with cardiac pacing. At the time of first PCNL, he was not on any aspirin or other blood thinners. He started to take low-dose aspirin afterward, when cardiac pacing was placed for the treatment of sick sinus syndrome. His prior surgeries included right SWL and right PCNL performed in another institution, which did not totally solve the problem of the lithiasis. No other relevant pathologies emerged from his medical history and no previous metabolic work-up was carried out. Preoperative renal function did not appear compromised.

### Physical examination

He was 161 cm tall and weighed 84 kg; his BMI was 32.4 kg/m^2^. The abdomen was globus for adiposity. Blood pressure was normal as the other remaining vital signs. No remarkable skeletal abnormalities emerged.

### Diagnostic studies

**FIG. 1.**
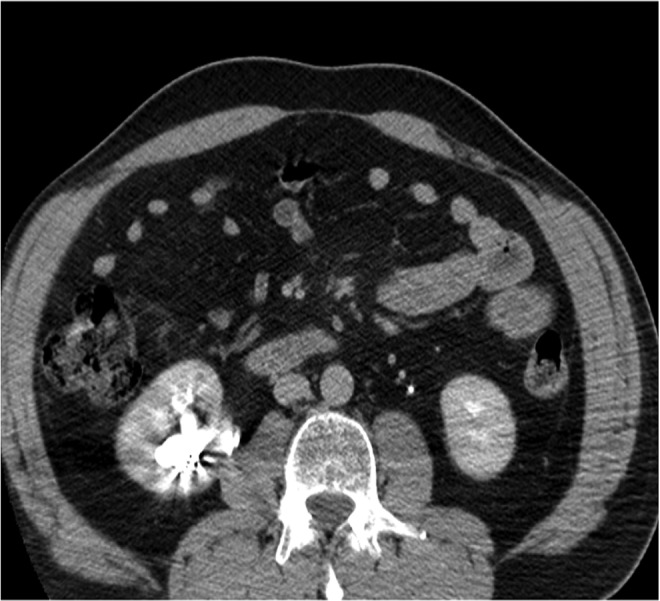
Preoperative CT scan.

**FIG. 2.**
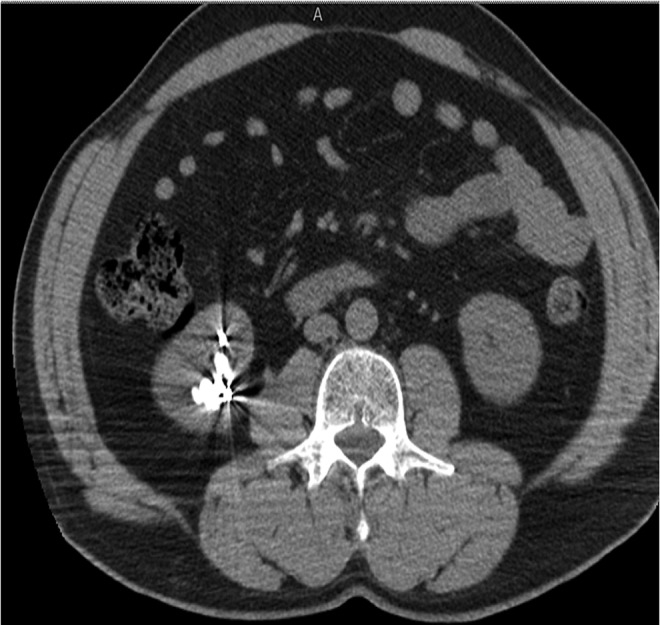
Preoperative CT scan.

**FIG. 3.**
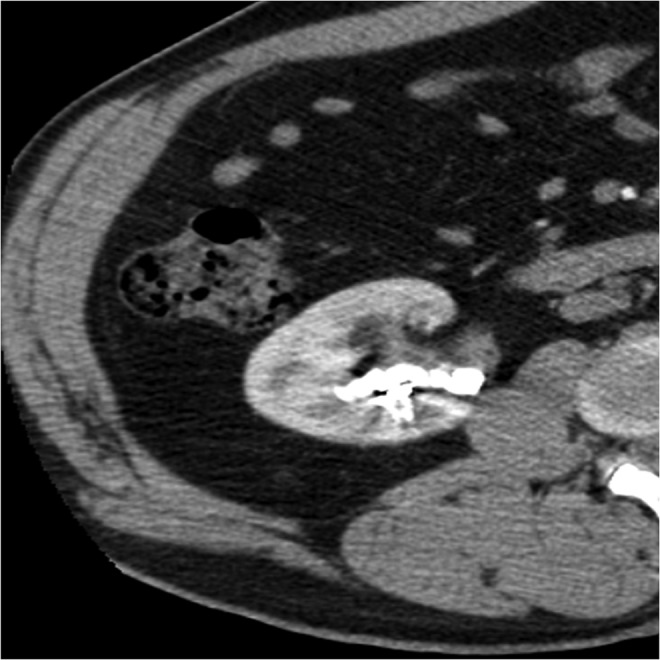
Preoperative CT scan showing metal coils of previous SRAE. SRAE, selective renal artery embolization.

Preoperative noncontrast CT scan revealed multiple and large stones involving renal pelvis and the upper, middle, and lower caliceal system with mild dilatation of the urinary tract (Figs. 1 and 2) and metal coils of SRAE (Fig. 3). Greater stone fragment measured about 20 mm, Hounsfield Units were 955. Infundibulopelvic angle (measured as inner angle formed at intersection of ureteropelvic axis and central axis of lower pole infundibulum) gauged 67° and overall stone burden (length × width × 0.25 × 3.14) was about 815 mm^2^. Preoperative MAG-3 renal Lasix scan showed split function of 54.39% on the left and 41.65% on the right with decreased drainage on the latter side. Low-dose aspirin was replaced with low-molecular weight heparin 6 days before surgery. Urine analysis and urine culture were negative. Other laboratory parameters did not show any pathologic findings.

### Intervention

**FIG. 4.**
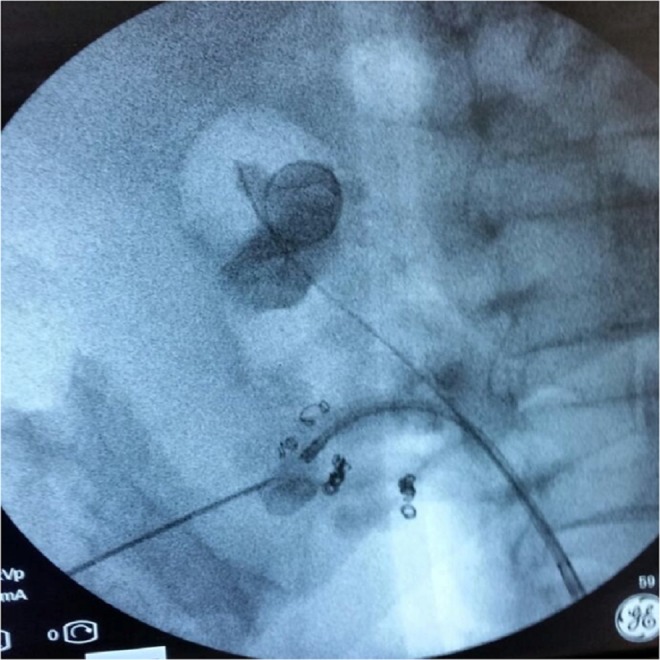
Lower-posterior kidney calix puncture under biplanar fluoroscopic and ultrasound guidance with also the additional assistance of the Endovision technique.

Double-J ureteral stent was previously placed to manage acute renal colic and hydronephrosis. ECIRS procedure was planned and performed after a few weeks by the same first surgeon who was already experienced in prone and supine PCNL, RIRS, and ECIRS. The patient was placed in Galdakao-modified supine Valdivia position combining the supine position of the patient with the flank elevated and a modified lithotomic arrangement of the lower limbs, the ipsilateral one extended and the contralateral one well abducted.^1^ Irrigant fluid (0.9% sodium chloride) was located at 50 cm above patient level to avoid high intraluminal pressures. Cystoscopy was performed using 22F cystoscope (Karl Storz-Endoskope^®^); retrograde pyelography, conducted with ureteral catheter 6F (RUSH^®^) and integrated by fluoroscopic guidance, which excluded the presence of ureteral strictures, malformations, or stone fragments, confirmed the stone characteristics previously evaluated by CT scan. A 0.035" hydrophilic guide (Boston Scientific Sensor^®^) was placed into the ureteral lumen. Subsequently, semirigid ureteroscopy was carried out using 8F ureteroscope (Karl Storz-Endoskope^®^) using a second guidewire. A 10- to 12-mm ureteral sheath (Coloplast Re-Trace^®^) was then located and flexible renoscopy with 8F flexible ureteroscope FLEX-X^2^ (Karl Storz-Endoskope) was conducted. Percutaneous renal access was carried out puncturing the lower-posterior kidney calix with Chiba-needle 18G under biplanar fluoroscopic and ultrasound guidance with also the additional assistance of the Endovision technique (Fig. 4). In view of the previous intervention and complication, this choice allowed us to sting the lower calix more precisely and safely as possible. Intraoperative urine samples for cultures from the upper urinary tract were systematically obtained. Upon insertion of guidewire 0.035" (BARD Black wire/ultra torque^®^) through the 18-guage needle sent down the ureter and the bladder, and exiting through the external meatus, percutaneous tract was dilatated to 24F using balloon (BARD X-Force^®^) and then Amplatz working sheath 24F was located. Stones were disintegrated using ballistic energy, with combined ballistic and ultrasonic (SWISS LITHOCLAST^®^ MASTER) or ballistic and Holmium laser energies (DORNIER Medilas H20^®^). We used Nephroscope 22F (Karl Storz-Endoskope) to ensure a good outflow of the irrigation liquid between the Amplatz sheath and the nephroscope during the procedure. Stone fragments were extracted using Nitinol basket 1.9F (Zero Tip^®^ Boston Scientific) and extractors 10F (Perc N-Circle^®^ COOK). At the end of the procedure, a Double-J ureteral stent 6F (Polaris Ultra Boston Scientific^®^) and nephrostomy tube 8F (Soft Drain Bard^®^) were inserted. Postoperative pyelography did not show any contrast leakage outside the collecting system and outside the ureter after the removal of the ureteral access sheath and no evident signs of residual stone fragments. Operative time was about 50 minutes.

### Outcome

**FIG. 5.**
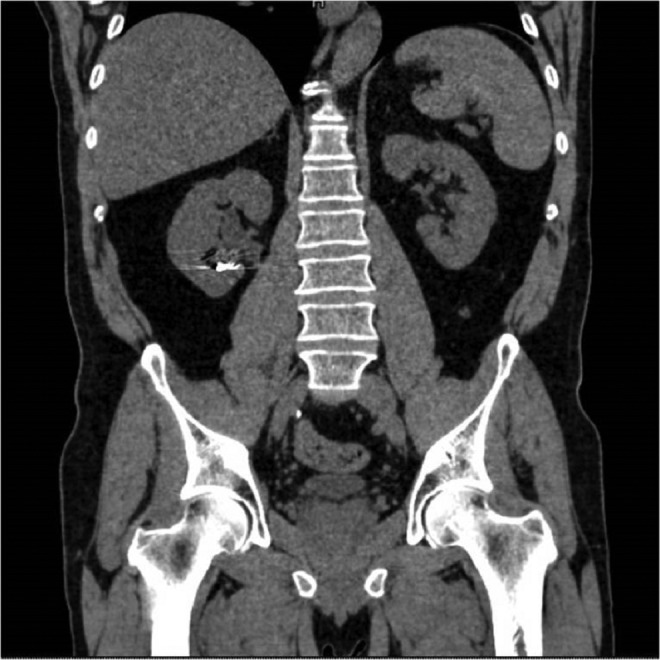
One-year postoperative CT scan showing nonresidual stone fragments, but only residual metal coils of previous SRAE.

Postoperative laboratory studies showed stable levels of hemoglobin, hematocrit, platelets, electrolytes; renal function, evaluated with creatinine and blood urea nitrate, revealed preserved. CT scan 2 days and 1 year after PCNL demonstrated a complete stone-free rate and no stone recurrence, showing only residual metal coils of previous SRAE (Fig. 5). Nephrostomy tube was removed 5 days after surgery, always preceded by a pyelography checkup that did not show any residual stone fragment. Renal scintigraphy performed few months after surgery, to assess a possible renal function impairment, showed results similar to those of the preoperative. Finally stone composition analysis reported mixed fragments of uric acid and calcium oxalate and metabolic work-up pointed out high levels of uric acid, sodium, and calcium and low levels of citrate in the urinary parameters. Therefore specific and general preventive measures were taken to prevent urolithiasis recurrence in the patient.

## Discussion and Literature Review

Salvage PCNL is defined as PCNL performed on patients, referred from another provider after an initial unsuccessful attempt, that was made to treat an upper tract stone using a percutaneous access.^2^ Unsuitable access to the stone is the most common reason for prior failed attempt with 80% of salvage procedures associated with prior difficulty with accessing and treating the stone. Other reasons include infection (hemodynamic instability in the presence of purulent urine) and excess bleeding. Access related to complications during PCNL includes renal hemorrhage, failed access, thoracic complications, and injury to other organ system. The risk of major renal hemorrhage requiring transfusion during percutaneous renal entry ranges from 1% to 3%^3^ and entering at the most peripheral point of the calix ensures minimization of wide hemorrhages requiring embolization. Many studies defined the learning curve for PCNL, jointly suggesting that at least 50 to 60 procedures are needed to acquire reliable competence in obtaining access to the kidney and a success rate between 82.5% and 97.6%.^4,5^ Although salvage PCNL data on its safety and cogency, after previous unsuccessful PCNL, are available,^2^ this is not yet for salvage ECIRS although its effectiveness has already been demonstrated as primary procedure.^6,7^ We wanted to provide ECIRS feasibility in a salvage setting after previous failed PCNL. Our results confirm that this is a safe and achievable approach also due to the possibility to perform a more precise puncture of the kidney combining ultrasound and two-dimensional fluoroscopy imaging with the Endovision technique. It ensures no relevant complications, a complete stone-free rate, and a disease-free recurrence after 1 year of follow-up. Finally, worthy of note is that in these patients, where the complexity of urolithiasis is often linked to metabolic disorders, a metabolic work-up should always be considered to achieve the best result.

## Conclusion

To our knowledge, this is the first case of salvage ECIRS reported in literature after previous PCNL. Even after SRAE, this procedure appears as safe and as efficacious as standard salvage PCNL when performed by experienced hands.

## Abbreviations Used

BMI: body mass index
CT: computed tomography
ECIRS: endoscopic combined intrarenal surgery
PCNL: percutaneous nephrolithotomy
RIRS: retrograde intra renal surgery
SRAE: selective renal artery embolization
SWL: extracorporeal shockwave lithotripsy
